# Microbiological Status of Grass Silages in Relation to Agricultural Practices: A Four-Year Study

**DOI:** 10.3390/foods15173060

**Published:** 2026-08-29

**Authors:** Elżbieta Kukier, Łukasz Bocian, Monika Pytka

**Affiliations:** 1 School of Medical & Health Sciences, VIZJA University, 59 Okopowa Street, 01-043 Warsaw, Poland; 2 Department of Research Support, National Veterinary Research Institute, 57 Partyzantów Avenue, 24-100 Pulawy, Poland; lukasz.bocian@piwet.pulawy.pl; 3 Department of Biotechnology, Microbiology and Human Nutrition, University of Life Sciences, Akademicka 13, 20-950 Lublin, Poland; monika.pytka@up.edu.pl

**Keywords:** grass silage, microbial safety, foodborne pathogens, *Clostridium*, *Listeria*, *Bacillus*, *Salmonella*, *Campylobacter*, silage pH

## Abstract

The microbiological quality of silage, a fundamental component of cattle feed, is critical to both animal health and human safety. This long-term study evaluated the microbiological status of grass silages collected from Polish cattle farms, aiming to assess the prevalence of zoonotic agents and hygiene indicators in relation to pre-ensiling and ensiling agricultural practices. A total of 160 grass silage samples were randomly collected nationwide by veterinary officers across various municipalities. The researchers measured pH values and conducted analyses to determine the presence and loads of pathogens, including *Listeria* spp., *Clostridium* spp., *Salmonella* spp., *Escherichia coli*, and *Bacillus cereus*. Only 17.5% of grass silages fell within the optimal target pH range. Conversely, 80.6% exceeded the upper reference threshold, indicating widespread poor fermentation and low silage quality. *Listeria* species were detected in 5.6% of the forages, occurring exclusively in silages with an incorrect pH. *Clostridium* spp. were highly prevalent, testing positive in 82.5% of all samples, while *C. perfringens* (predominantly toxotype A) was identified in 26.2%. Both *Salmonella* spp. and botulinum neurotoxin-producing *Clostridia* were detected in separate, single samples, both characterized by high pH levels. Additionally, fungal counts reached high, non-feedable levels exceeding 6 log_10_ cfu/g in 10.6% of the tested silages. Silage inoculation, practiced by 26.2% of farmers, significantly lowered pH values and reduced the counts of spoilage microorganisms. Additionally, inoculated silages had a more than three-fold greater likelihood of achieving the correct pH. In contrast, organic fertilization was associated with an increase in the mean counts of *Clostridium* spp. and *B. cereus*. In conclusion, grass silage is highly vulnerable to poor fermentation and microbial contamination.

## 1. Introduction

Currently, roughage accounts for approximately two-thirds to three-quarters of a ruminant’s diet. Grass silage (GS) is the most common roughage worldwide after maize silage (MS), comprising up to one-third of the total feed dry matter consumed by ruminants [1]. In Poland, GS is a staple of cattle nutrition, reflecting a growing cattle population and a dairy herd that currently ranks as the third largest in the European Union [2]. This forage consists of young grasses preserved through fermentation during ensiling. A key advantage of GS over MS is its higher protein and antioxidant content [3]. Good-quality GS is characterized by high nutritional value, aerobic stability, low pH, excellent intake potential, and microbiological safety [4]. Despite extensive data on the nutritional value of GS, information regarding the microbial quality of good-quality GS remains limited [5,6,7]. The microbial load of ready-to-feed silage is influenced by the composition of epiphytic microflora on the standing crop, secondary contamination during ensiling, and conditions during storage and feed-out [8,9].

From a food chain hygiene perspective, GS may act as a source or vector of soil-borne microorganisms, particularly when the pH is not sufficiently reduced or when oxygen is present. While some agents, such as yeasts, primarily impair nutritional value, others pose a significant hazard to animal health and the safety of food of bovine origin. Of particular concern is the presence of pathogens from the *Listeria*, *Clostridium*, and *Bacillus* genera, members of the *Enterobacteriaceae* family, and toxigenic fungi [10]. Microbial proliferation is favored by the high water activity of silage, its buffering capacity, and the presence of water-soluble carbohydrates [11]. Anaerobic conditions, combined with the fermentation end-products of lactic acid bacteria, lower the final pH and inhibit undesirable microorganisms, thus acting as natural preservatives [12]. However, if the ensiling process does not proceed correctly, spoilage or pathogenic microbes may proliferate, making the GS microbiologically hazardous [13]. Certain grasses, such as meadow grass or fescue, are more challenging to ensile due to the lower water-soluble carbohydrate content of the fresh material. Additional challenges in GS production include aerobic conditions resulting from inadequate sealing or low moisture levels. Particular attention is paid to grasses harboring viable pathogens from organic fertilizers or endemically contaminated soil, as well as their ability to survive and proliferate during the ensiling process [14].

Moreover, exposing animals to feed with a high microbial burden stimulates the immune system. Excessive immune activation triggers the production of pro-inflammatory cytokines and interferons, initiating an acute-phase response. This cascade leads to fever, inappetence, and muscle amino acid resorption, while nutrient partitioning is redirected from meat and milk production toward hepatic synthesis of acute-phase proteins and leptin [15]. Consequently, catabolism outweighs anabolism, leading to reduced animal productivity and profitability.

Furthermore, to ensure animal and human safety, zoonotic agents are monitored in Europe under Directive 2003/99/EC across all relevant stages of the food chain, including primary production. Given these considerations, this study aimed to evaluate the microbial status of GS derived from cattle farms, specifically assessing the prevalence of zoonotic agents in relation to pre-ensiling and ensiling factors—such as farm characteristics, grassland cultivation practices, and the ensiling process. We hypothesized that specific agricultural practices significantly influence the hygienic quality of GS.

## 2. Materials and Methods

### 2.1. Experimental Design and Sampling

**Figure 1:**
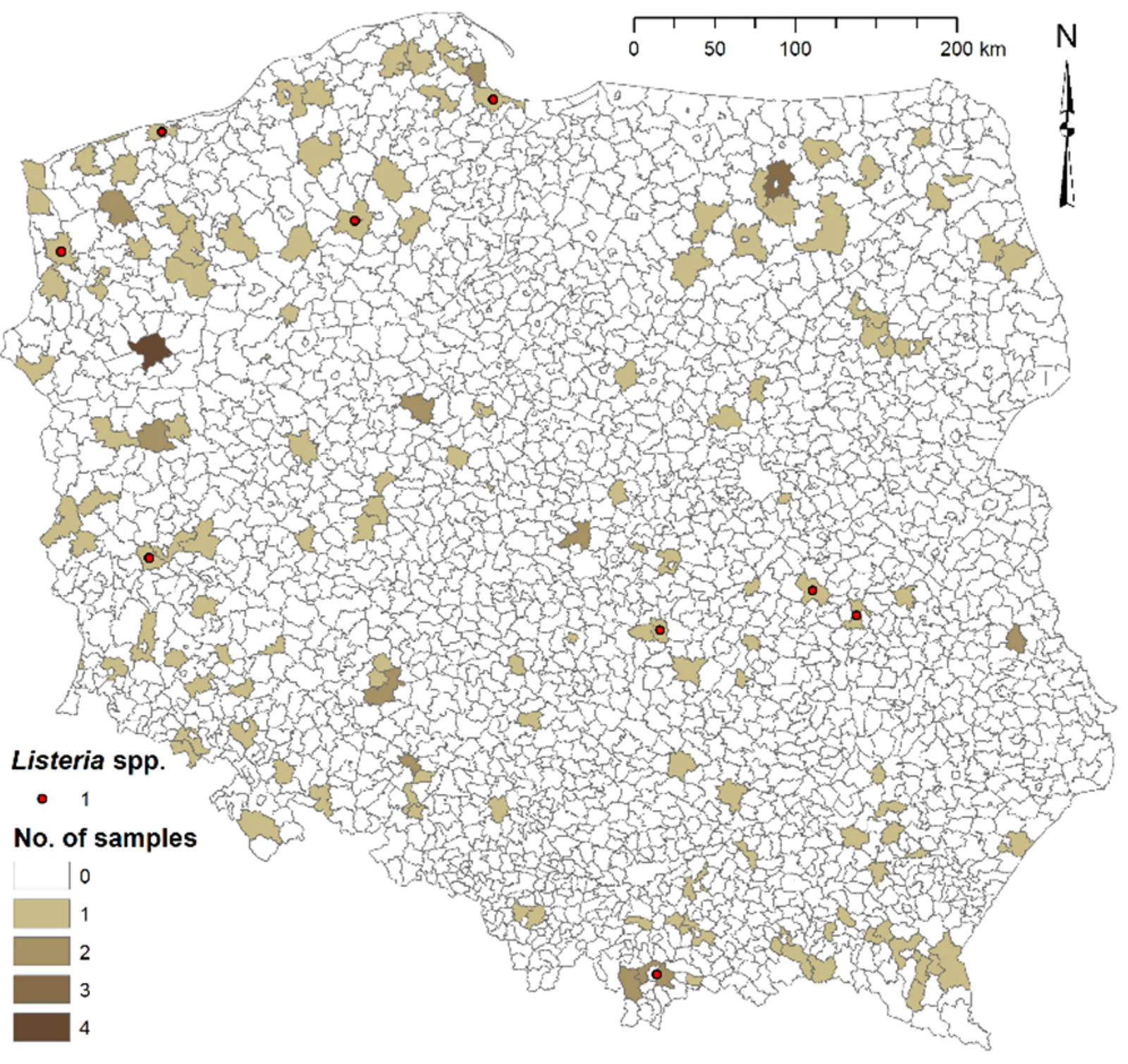
Geographical distribution of GS samples across Polish municipalities, with background color intensity reflecting the number of samples per municipality. Red dots mark locations where *Listeria* spp. were isolated.

A total of 160 GS samples were collected from Polish cattle farms between 2014 and 2017 (n = 50, 32, 39, and 39, respectively). Random nationwide sampling was performed annually from January to November by veterinary officers, who collected between two and four samples per voivodeship. The distribution of these samples across individual municipalities is illustrated in Figure 1. To minimize oxygen exposure, GS samples were obtained from the bale core using a hay core sampler after removing the surface layer and any visible mold and placed into sterile plastic bags. Aseptic techniques were strictly maintained to prevent secondary contamination, in line with good microbiological practices. Following collection, samples were immediately frozen and transported on ice to the National Veterinary Research Institute (NVRI) in Puławy, Poland, and maintained at −20 °C for a period of up to 7 days [16]. Samples were thawed at 2–8 °C for at least 24 h prior to analysis. Each silage was accompanied by a detailed questionnaire regarding farm characteristics, grass cultivation practices, and ensiling procedures. Specifically, the data collected encompassed cattle herd size, the farmer’s experience in forage ensiling, soil quality, and the application of herbicides or organic fertilizers. Additionally, information on the use of silage inoculants, silage maturity, and the farm’s history of zoonotic diseases, such as listeriosis and botulism, was recorded.

### 2.2. Measurement of pH Value

For pH measurement, 100 g of GS and 100 mL of double-distilled water were placed in a sterile filtered stomacher bag and homogenized for 2 min using a stomacher (MIX2, AES Laboratoire; Combourg, France). After overnight storage at 2–8 °C, the samples were allowed to reach ambient temperature for one hour before analysis. At the beginning of the analysis, the electrode bulb was rinsed with distilled water, and the pH meter (CG 843P, SCHOTT; Mainz, Germany) was calibrated using standard buffer solutions of pH 4.0 and 7.0. The pH of each filtrate was then recorded to assess fermentation quality, with a reference range of 3.8 to 4.4 used for GS [17].

### 2.3. Microbiological Analysis

The presence of *Salmonella* spp., *Clostridium* spp., *L. monocytogenes*, and *Campylobacter* spp. was detected using qualitative standard culture methods [18,19,20,21]. The counts of total plate count (TPC), aerobic mesophilic bacteria (AMB), fungi, anaerobic spore-forming bacteria (ASFB), *Enterobacteriaceae* family, *Escherichia* (*E.*) *coli*, presumptive *Bacillus* (*B.*) *cereus*, *Clostridium* (*C.*) *perfringens*, mesophilic lactic acid bacteria (LAB), and coagulase-positive staphylococci (CoPS) were estimated according to quantitative standard culture methods [22,23,24,25,26,27,28,29]. Standard culture methods for this survey follow the protocols described in our previous study [30]. Preparation of silage samples, initial suspensions, and decimal dilutions followed ISO 6887-4:2017 [31]. Experimental conditions were validated through duplicate quantitative analyses and the inclusion of reference-strain-based controls.

### 2.4. Typing of Clostridium Isolates by PCR Methods

All strains of *C. perfringens* isolated from forages were toxotyped with a multiplex polymerase chain reaction (mPCR), where the presence of *cpa* (α toxin), *cpb* (β), *cpb2* (β2), *etx* (ε), *iap* (ι), and *cpe* (enterotoxin) toxin genes was detected according to the protocol by Baums et al. [32]. The extraction of DNA was slightly modified since the template DNA was taken from a thermolysed overnight culture of *C. perfringens* on Willis-Hobbs agar after incubation at 37 °C in anaerobic conditions [33]. Control strains of known bacteria were included in the extraction procedure. Isolates suspected to be botulinum neurotoxin (BoNT)-producing *Clostridia* were confirmed by a real-time PCR method in which the nontoxic nonhemagglutinin encoding gene (*ntnh*), distinctive to all BoNT-producing *Clostridia*, was detected [34]. Genomic DNA extraction was conducted with the Genomic MINI AX Bacteria (A&A Biotechnology, Gdańsk, Poland) following the manufacturer’s protocols. The LightCycler 2.0 System (Roche, Rotkreuz, Switzerland) was used for the real-time PCR method. The toxotypes of BoNT-producing *Clostridia* were identified in the thermocycler T-1 Thermoblock (Biometra, Göttingen, Germany) with the mPCR technique [35].

### 2.5. Identification of Bacteria Using MALDI-TOF MS

Other questionable bacteria were identified by matrix-assisted laser desorption/ionization time-of-flight mass spectrometry (MALDI-TOF MS) using a Bruker MALDI Biotyper (Bruker, Bremen, Germany) according to the manufacturer’s ethanol-formic acid extraction protocol. The system was calibrated using the Bacterial Test Standard (Bruker Daltonics). All identification steps followed the procedure previously described for isolates from maize silage [30]. Each isolate was tested in duplicate, and the highest score was recorded. In this study, score values of ≥2.0 and ≥1.7 were taken as the cut-off values for species- and genus-level identification, respectively. Bacteria identified with score values below 1.6 were considered unreliable.

### 2.6. Calculations and Statistics

Qualitative microbial results were expressed as percentage prevalence. Quantitative counts, measured in colony-forming units per gram (cfu/g), were log_10_ transformed prior to statistical analysis; no constant was added as all values exceeded zero. All samples meeting the inclusion criteria were retained, with no outliers excluded. *Clostridium* spp. presence was determined by titer (the minimum weight of sample in grams where microorganism presence was confirmed). For graphical representation, these titers were converted into microbial ranges: a titer of <0.1 indicated <10 cfu/g (below 1 log_10_), a titer of 0.1 represented 10–99 cfu/g (1 log_10_), and a titer of 0.01 corresponded to 100–999 cfu/g (2 log_10_), continuing accordingly. Microbial loads were evaluated in silages with both correct and incorrect pH values. Furthermore, the impact of annual atmospheric conditions in Poland was incorporated, specifically mean annual rainfall (2014: 644.3 mm; 2015: 501.2; 2016: 701.2; 2017: 794.16) and mean annual temperatures (2014: 8.4 °C; 2015: 8.4; 2016: 8.1; 2017: 7.75).

Data normality was assessed using the Shapiro–Wilk test. Due to the non-normal distribution of quantitative variables, the non-parametric Mann–Whitney U test was employed for comparisons, while qualitative parameters of grass silages were analyzed using χ^2^ tests. For analyses involving binary variables, effect size was expressed as odds ratios (ORs) with corresponding 95% confidence intervals (CIs), where applicable. For multi-year comparisons, the Kruskal–Wallis test with a post hoc multiple comparison test was applied. The strength and direction of associations between farm characteristics and silage microbial parameters were determined using Spearman’s rank correlation coefficient (R). Absolute R values were categorized as weak (0.1–0.3), moderate (0.3–0.5), or strong (0.5–1.0). Statistical significance was set at ≤0.05 for all analyses. Data processing was performed using TIBCO Statistica, version 13 (TIBCO Software Inc., Palo Alto, CA, USA). The geographical distribution and spatial visualization (Figure 1) were generated using ArcGIS Desktop 10.4.1 (Esri Inc., Redlands, CA, USA).

## 3. Results

### 3.1. Farm Characteristics

The study included GS samples collected from farms varying in size from 5 to 1693 animals. The experience of farmers in grass ensiling ranged from one to approximately 60 years. The green fodder was collected from meadows spanning soil quality classes I–VI, categorized as follows: I (excellent), II (very good), III (good), IV (medium), V (poor), and VI (very poor). Organic fertilization was practiced on 44.4% (71/160) of farms, primarily using manure or slurry (43.1%; 69/160) and poultry litter (1.2%; 2/160). None of the meadows received digestate from biogas plants. Herbicide application for weed control in meadows was reported on 13.1% (21/160) of the surveyed farms. Inoculation was applied to 26.2% (42/160) of GSs, with annual rates of 28% in 2014, 15.6% in 2015, 30.7% in 2016, and 28.2% in 2017. At the time of sampling, silage maturity ranged from approximately 1 to 16 months. Farmers reported all GS batches as safe, with no clinical disease symptoms attributed to poor silage quality observed immediately prior to sampling. However, survey data showed a history of listeriosis at one cattle farm (0.6%) and botulism at another (0.6%). Additionally, none of the sampled GSs exhibited visible mold infestation.

### 3.2. pH of Grass Silages

The pH of the silages ranged from 3.2 to 6.8. Only 17.5% (28/160) of samples fell within the target range, while 82.5% were outside optimal limits. Specifically, 1.9% (3/160) of silages were below the lower reference threshold, and 80.6% (129/160) exceeded the upper limit. Analysis showed that 3.7% of GSs had a pH below 4, 35.6% ranged from pH 4 to 5, and 60.6% had a pH above 5.

### 3.3. Prevalence of Bacteria

*Listeria* species were detected in 5.6% (9/160) of the surveyed forages. While samples with optimal pH were free of these bacteria, *Listeria* spp. were detected in 6.8% (9/132) of silages with incorrect pH. The pH of *Listeria*-positive samples ranged from 4.5 to 6.6. The overall prevalence of individual *Listeria* species was 2.5% each for *L. monocytogenes* and *L. innocua* and 0.6% for *L. seeligeri*. Among the nine isolates identified, *L. monocytogenes* and *L. innocua* each accounted for 44.4% (n = 4), while *L. seeligeri* represented 11.1% (n = 1) of the total. Of the total silages, 82.5% (132/160) tested positive for *Clostridium* spp., comprising 78.6% (22/28) of samples with correct pH and 83.3% (110/132) of those with incorrect pH. *C. perfringens* was identified in 26.2% (42/160) of all samples, occurring in 17.8% (5/28) of correct pH forages and 28% (37/132) of those with incorrect pH. BoNT-producing *Clostridia* were detected in a single silage (0.6%) with a pH of 5.4. This sample exhibited a *Clostridium* spp. level of log_10_ cfu/g, while both *C. perfringens* and *Listeria* spp. were absent. The farm of origin, which maintained a herd of 35 cattle and had produced GS for five years, had no recorded history of botulism or listeriosis. Furthermore, no poultry litter or digestate had been applied as fertilizer during the growing seasons prior to ensiling. *Salmonella* was detected in a single sample (0.6%) with a pH of 6.4, showing contamination levels of 1 log_10_ cfu/g for both *Enterobacteriaceae* and *E. coli*. The *Salmonella*-positive forage originated from a meadow fertilized with manure on a farm with 95 cattle, where GS had been produced for ten years. Notably, neither the BoNT-producing *Clostridia*-positive silage nor the *Salmonella*-positive sample had been treated with inoculants during production. *Campylobacter* spp. were not detected in any of the tested GS samples. The overall prevalence of *Enterobacteriaceae*, *E. coli*, and *B. cereus* was 10.6%, 5.6%, and 50.6%, respectively. Lower prevalence rates were observed in silages with correct pH (0%, 0%, and 50%), whereas higher rates occurred in incorrect pH forages (12.9%, 6.8%, and 50.7%). In addition to *B. cereus*, three other species were identified: *B. weihenstephanensis*, *B. mycoides*, and *B. thuringiensis*. Coagulase-positive staphylococci were not found in any of the analyzed forages.

### 3.4. Toxotypes of Clostridium Isolates

The toxotypes of A (95.2%), D (2.4%), and E (2.4%) among the total of 42 *C. perfringens* isolates were detected. *C. perfringens* isolates from correct pH silage were assigned as type A (100%), and strains isolated from incorrect pH silage were classified as type A (94.6%), D (2.7%), and E (2.7%). The presence of the *cpb2* gene was shown in 45.2% of the total *C. perfringens* isolates, including 20% of isolates from GSs of correct pH and 48.6% of isolates from incorrect pH forages. The *cpb2* gene was demonstrated in strains of type A (42.5%), D (100%), and E (100%). The isolated BoNT-producing *Clostridium* strain was identified as *C. botulinum* type B.

### 3.5. Count of Hygiene Indicators

The distribution of microbial counts and descriptive statistics for GSs with a correct pH are summarized in Table 1. The mean TPC and AMB counts in GSs with correct pH values were similar, and the upper TPC limit was higher in only a few individual GSs. Fungal contamination was skewed toward the lower limit of the range. In 67.8% of the analyzed forages, fungal counts were restricted to 1 log_10_ cfu/g, resulting in low mean and median values. The LAB counts exhibited a wide distribution range, with 50% of the samples maintaining high populations. Average contamination levels of *Enterobacteriaceae*, *E. coli*, *C. perfringens*, and *B. cereus* were similar, though the upper limit was slightly lower for *C. perfringens* and higher for *B. cereus*. The genus *Clostridium* was detected across a wide range, whereas *C. perfringens* was highly restricted, with 92.8% of the samples remaining at 1 log_10_ cfu/g. Median values were comparable to the means for TPC, LAB, *Enterobacteriaceae*, and *E. coli*; however, they were lower for AMB, fungi, *C. perfringens*, and *B. cereus* and higher for the *Clostridium* load.

Silages with incorrect pH values demonstrated a higher microbial burden compared to those with correct pH. This trend was reflected in both the mean values (increases of up to 1 log_10_ cfu/g) and the upper limits of the microbial ranges (reaching 5.3 log_10_ cfu/g). The mean counts of *E. coli* and *C. perfringens* remained stable across forages, regardless of pH status. Significant differences were observed in the distribution of silages across individual microbial levels, as well as in the percentage of silages at specific contamination levels for the enumerated microorganisms. In silages with incorrect pH, median values were comparable to the means for TPC, AMB, and *E. coli* but lower for fungi, *Enterobacteriaceae*, *C. perfringens*, and *B. cereus* and higher for LAB and ASFB load. The distribution of microbial counts and descriptive statistics for GSs characterized by an incorrect pH value are presented in Table 2.

The microbiological status of the total silage samples showed significant variation across the different microbial groups. The majority of forages clustered between 3 and 7 log_10_ cfu/g in terms of overall bacterial load, as represented by TPC and AMB. Both groups exhibited the widest distribution ranges, occasionally exceeding 9 log_10_ cfu/g. Lactic acid bacteria followed a similar distribution pattern to TPC and AMB but with a notable concentration of samples (27.5%) peaking at 6 log_10_ cfu/g. The close alignment of mean and median values for LAB presents a relatively symmetrical distribution and confirms their dominant role in the samples’ core microbiota. In contrast, fungal contamination was substantially lower. Nearly half of the samples (46.9%) contained fungi at the lowest detection level (1 log_10_ cfu/g), and counts above 7 log_10_ cfu/g were negligible. This was reflected in the significantly lower mean and median values compared to the total aerobic counts. Indicator organisms and potential pathogens showed very low prevalence. *Enterobacteriaceae* and *E. coli* were absent or at minimal levels in the vast majority of samples (90.6% and 98.8%, respectively). Similarly, while *Clostridium* species were detected across a moderate range (1–5 log_10_ cfu/g), *C. perfringens* and *B. cereus* were largely confined to the lowest concentrations. For these three groups, the median values remained at the detection limit (1.0 log_10_ cfu/g), highlighting that significant contamination was the exception rather than the rule. The distribution of forages across different microbial levels in the total GS population is presented in Table 3.

The qualitative tests showed a significant association between GSs of correct pH and both inoculation (*p* = 0.0016) and herbicide application (*p* = 0.0077). Furthermore, quantitative analyses demonstrated statistically significant differences in silages of correct pH regarding the counts of AMB (*p* = 0.0257), fungi (*p* = 0.0145), LAB (*p* = 0.0300), and *Enterobacteriaceae* (*p* = 0.0461), as well as herd size (*p* = 0.0107) and ensiling experience (*p* = 0.000047), compared to GSs of incorrect pH. The TPC was close to the significance threshold (*p* = 0.0592). Other results were not statistically significant. The comparison of results across individual years showed statistically significant differences in fungal counts between 2014 and 2017 (*p* = 0.0282), and in LAB counts between 2014 and 2017 (*p* = 0.0365). Regarding *B. cereus* contamination, significant differences were observed between 2014 and 2016 (*p* < 0.0001) as well as between 2016 and 2017 (*p* = 0.0018). Moreover, the analysis indicated significant associations in average annual rainfall among all tested years (*p* < 0.0001). For mean annual temperature, significant differences were found between 2014 and 2015 versus 2016 (*p* < 0.0001) and 2017 (*p* < 0.0001). Specifically, significant variations were noted for 2016 versus 2014 (*p* < 0.0001), 2015 (*p* < 0.0001), and 2017 (*p* = 0.0012), as well as for 2017 versus 2014 (*p* < 0.0001), 2015 (*p* < 0.0001), and 2016 (*p* = 0.0012). No statistically significant differences were observed for the remaining year-to-year comparisons.

### 3.6. Effect of Organic Fertilization

Organically fertilized grasses yielded GSs with pH values ranging from 3.20 to 6.82 (mean 5.22, median 5.38), while non-fertilized grasses produced pH values between 3.60 and 6.80 (mean 5.16, median 5.25). In organically fertilized GSs, the prevalence of bacteria was 4.2% for *Listeria* spp., 84.0% for ASFB, 31.0% for *C. perfringens*, 4.2% for the *Enterobacteriaceae* family, 2.8% for *E. coli*, and 52.1% for *B. cereus*. In non-fertilized GSs, these parameters were estimated at 6.7%, 80.9%, 22.5%, 15.7%, 7.9%, and 49.4%, respectively. Additionally, an outbreak of botulism (1.4%) and one outbreak of listeriosis (1.4%) occurred on farms using organic fertilization, whereas these were not diagnosed on farms without organic fertilization.

The mean loads of TPC, fungi, LAB, *Enterobacteriaceae*, and *E. coli*, along with the median values for TPC, fungi, and LAB, and the upper limits for TPC, fungi, LAB, and *E. coli*, were higher in GSs originating from non-fertilized grasses. Conversely, organic fertilization was associated with an increase in the mean counts of AMB, *Clostridium* spp., and *B. cereus*. Grass silages produced from organically fertilized grasses showed statistically significant differences in *Enterobacteriaceae* counts (*p* = 0.0237) and herd size (*p* = 0.0192).

### 3.7. Effect of Silage Inoculation

Inoculated GSs demonstrated pH values ranging from 3.20 to 6.30 (mean 4.81, median 4.80), while non-inoculated GSs ranged from 3.36 to 6.82 (mean 5.32, median 5.45). In inoculated GSs, 78.6% were *Clostridium*-positive, 9.5% were *Enterobacteriaceae*-positive, and 0.5% were *E. coli*-positive; in contrast, non-inoculated forages showed a prevalence of 83.9%, 11.0%, and 5.9%, respectively. An opposite trend was observed for *Listeria* spp., *C. Perfringens*, and *B. cereus*, with prevalence rates of 7.1%, 33.3%, and 52.4% in inoculated forages compared to 5.1%, 23.7%, and 50.0% in non-inoculated GSs. Inoculated silages showed lower means and medians for TPC, AMB, fungi, LAB, and ASFB, as well as a reduction in the upper limits for TPC, AMB, fungi, LAB, *E. coli*, *C. Perfringens*, and *B. cereus* compared to non-inoculated GSs. Furthermore, inoculated GSs showed statistically significant differences in herd size (*p* < 0.0001), fungal counts (*p* = 0.0148), and forage pH (*p* = 0.0017). The estimated odds ratio indicated that inoculation was associated with more than three-fold higher odds of achieving the correct silage pH (OR = 3.71; 95% CI: 1.59–8.69). The differences in microbial loads among inoculated, non-inoculated, organically fertilized, and non-fertilized silages are presented in Table 4.

### 3.8. Correlation of Parameters

**Figure 2:**
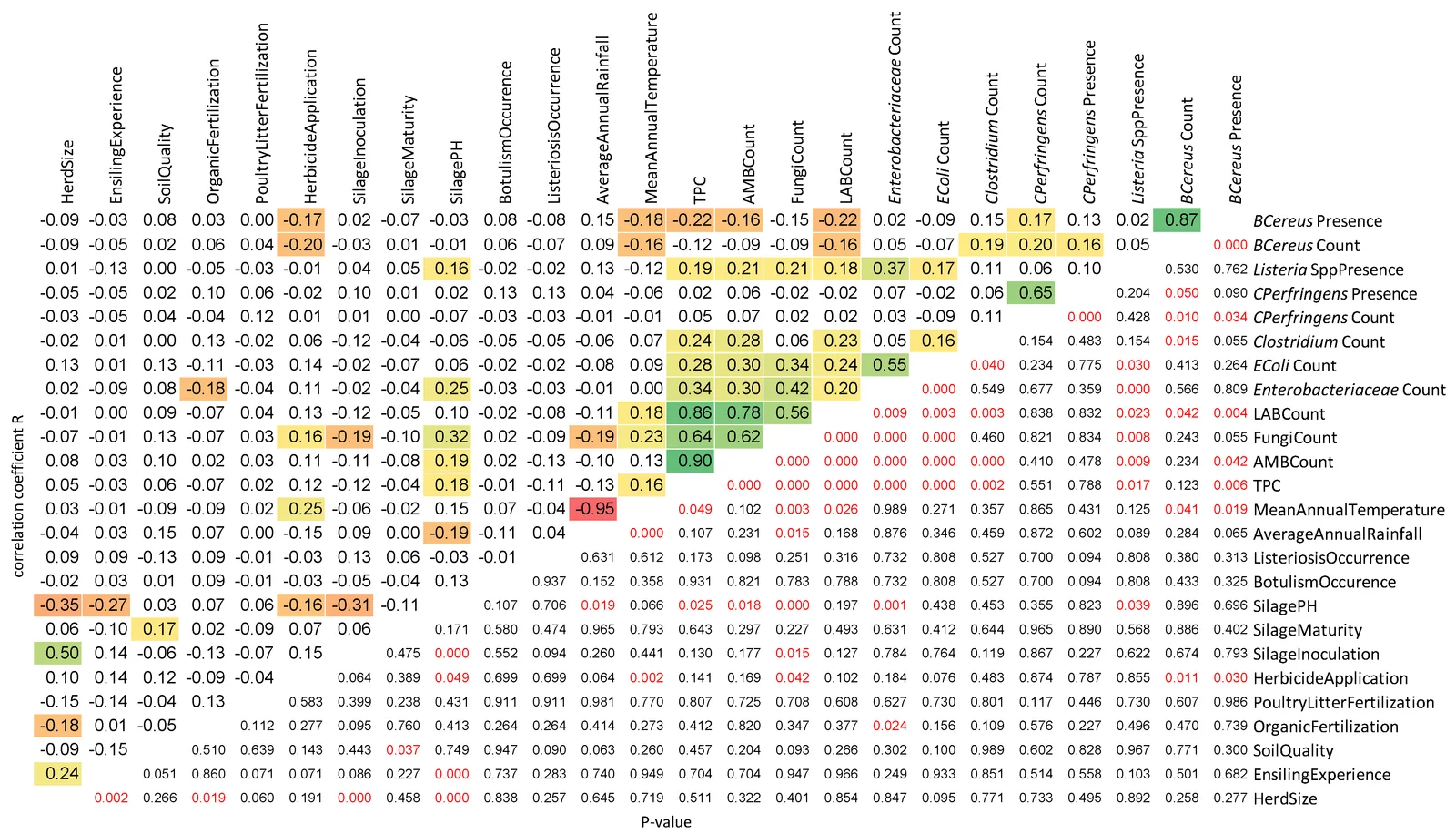
Spearman’s correlation matrix illustrating the relationships among all analyzed agricultural, environmental, and microbiological variables. Correlation coefficients are presented in the upper triangle, while the statistical significance of these correlations is displayed in the lower triangle. *p*-values marked in red indicate statistically significant correlations. Significant correlation coefficients are highlighted using a red–yellow–green color scale, ranging from −1 (negative correlation) to +1 (positive correlation).

Owners of larger herds and those with more ensiling experience applied inoculants more frequently (Figure 2). Inoculation was negatively correlated with fungal counts. Herd size, ensiling experience, silage inoculation, herbicide application, and average annual rainfall were all negatively correlated with the pH value of the GSs. Conversely, a positive association was observed between pH and TPC, AMB counts, fungal counts, the *Enterobacteriaceae* family, and *Listeria* spp. prevalence. The prevalence of *B. cereus* was weakly and negatively associated with herbicide application, mean annual temperature, TPC, AMB counts, and LAB counts. A strong negative correlation was demonstrated between mean annual temperature and average annual rainfall. Lower average annual rainfall corresponded to lower enumerated fungal counts. Higher mean annual temperatures were associated with increased TPC, fungi, and LAB counts. Strong or moderate positive associations were observed between TPC and AMB, fungi, LAB, *Enterobacteriaceae*, *E. coli*, and *Clostridium* counts, as well as *Listeria* spp. detection. Similar associations were estimated for AMB counts. Higher fungal counts were associated with increased LAB, *Enterobacteriaceae*, and *E. coli* counts and higher *Listeria* spp. prevalence. A positive correlation was found between LAB counts and the incidence of *Listeria* spp., as well as *Enterobacteriaceae*, *E. coli*, and ASFB loads. Higher *Enterobacteriaceae* counts corresponded with higher *E. coli* counts and *Listeria* spp. prevalence. A weak positive correlation was demonstrated between *E. coli* counts and the loads of *Clostridium* and *Listeria* spp. *Clostridium* spp., including *C. perfringens*, occurred more frequently when GSs were more heavily contaminated by *B. cereus*, and vice versa. Similarly, the *E. coli* load was positively linked to *Enterobacteriaceae*, ASFB, *Listeria* spp., LAB, and general hygiene indicator counts. The presence of *Listeria* spp. was favored by higher pH and increased levels of TPC, AMB, fungi, LAB, *Enterobacteriaceae*, and *E. coli*.

## 4. Discussion

The impact of feed on animal health and welfare, the safety of animal-origin foods, and human health is indisputable. Consequently, research into the microbiological status of silage and crop production practices affecting forage safety is essential. This approach enables the identification of critical points and the implementation of effective preventive measures to minimize hazards in primary production and downstream food products. Furthermore, it provides actionable insights for safer primary production practices in the future. To date, the microbiological status of GSs has typically been examined in the context of inoculation effects or epizootic investigations [36,37,38,39,40]. Consequently, knowledge regarding the microbial profile of GSs not associated with disease outbreaks remains limited. The random sampling of GSs across the country over four consecutive years, therefore, represents a unique and valuable aspect of this survey.

A significant concern in our study was the very high proportion of GS samples with an incorrect pH. This parameter serves as a reliable indicator of forage quality, fermentation, and preservation, as pH directly influences microbial survival in high-moisture roughage. The observed frequency of incorrect pH was considerably higher than that recorded for maize silages in our previous study [30]. Nearly all GS samples outside the reference range exhibited an excessively high pH, indicating poor fermentation and low silage quality, which leads to aerobic instability and secondary microbial growth [12]. The growth of undesirable bacteria is relatively slow at a pH of 4.5. Consequently, this indicator is used to classify silages into four categories: very good (pH 3.2–4.2), good (4.2–4.5), moderate (4.5–4.8), and poor (pH > 4.8) [41]. Based on this categorization, 10.6% of the tested GS samples were assessed as very good, 10.6% as good, and 11.9% as moderate, while the majority (66.9%) were classified as poor quality. For comparison, pH values for GSs reported in other studies have ranged from 3.5 to 8.2 [5,13,42,43,44,45,46,47,48,49,50,51,52,53,54,55]. The pH level is a key indicator of silage quality. Numerous reports indicate that the prevalence of zoonotic pathogens increases alongside rising silage pH [53,56]. According to a German evaluation based on the Deutsche Landwirtschafts-Gesellschaft (DLG) fermentation feed key, 24.9% to 44.7% of GSs showed poor fermentation quality. This study covered Lower Saxony, Schleswig-Holstein, Brandenburg, Mecklenburg-West Pomerania, Saxony-Anhalt, Thuringia, and Bavaria [48]. Among the German GS samples, only 7.9% had a pH below 4, while 81.8% fell between pH 4 and 5, and 10.2% exceeded pH 5. The observed fermentation quality of German GSs, where a significant portion of samples exceeded pH 5, aligns with the broader challenges identified in European forage production. Additionally, significantly more GSs were affected by poor fermentation quality (24.9–44.7%) than MSs (0–5%) in all German regions, which directly correlates with the increased risk of pathogen survival.

Since the *Listeria* genus is frequently found in soil and on vegetation, it has been suggested that these bacteria are a normal part of the microflora of grass. Big bale silages, in particular, appear to be highly susceptible to *Listeria* contamination due to their larger surface-to-volume ratio and a greater likelihood of aerobic surface spoilage. The low density and insufficient acidification of GSs favor the growth of facultatively anaerobic *Listeria* strains; indeed, pathogenic species of the genus have been identified in forages with excessively high pH values [57]. A positive correlation between the presence of *Listeria* species and silage pH was also confirmed in the monitored GSs. However, the recorded prevalence of *Listeria* spp. was lower than in other reports, where the proportion of *Listeria*-positive GS samples ranged from 25% to 60% [40,49,53,56,58]. Although the prevalence of *Listeria* spp. recorded in this study was lower than that reported by other researchers, the positive correlation between silage pH and the presence of these bacteria remains consistent with existing literature [58]. These results suggest that while local contamination levels may vary, maintaining a pH below 4 remains an effective strategy for inhibiting *Listeria* growth and ensuring the hygienic quality of grass silage. Even though *L. monocytogenes* can be introduced into the farm environment from various sources, poor-quality silage is considered the primary vehicle for contamination [56]. The ingestion of contaminated GS leads to either asymptomatic intestinal shedding or clinical mastitis in dairy cows, resulting in the direct excretion of *L. monocytogenes* into raw milk during milking. Furthermore, high pathogen loads in the gastrointestinal tract elevate the risk of carcass contamination via fecal shedding during the slaughter and skinning processes. Additionally, the biofilm-forming capacity of *L. monocytogenes* facilitates its transfer from farm environments to milking equipment and abattoirs, leading to the secondary contamination of meat and dairy products. To date, studies have reported a wide range of *L. monocytogenes* prevalence in grass silages, with recorded rates of 1.3%, 2.6%, 5%, 6%, 7.7%, and 17% [40,49,53,56,59,60]. The prevalence of *L. innocua* in other research was also higher than in our observations, reaching 11.5%, 19.3%, 26.3%, 40%, and 50% [40,53,56,59,60]. In contrast, the prevalence of *L. seeligeri* in our study was lower than the 1.2%, 4.76%, or 50% reported in the previous literature [40,56,61]. Furthermore, while some authors confirmed the presence of *L. monocytogenes*, *L. innocua*, and *L. seeligeri* in GSs [49,62], others additionally identified *L. welshimeri* and *L. grayi* species, which contrasts with our findings [56,59]. Regarding the distribution of species, a study of 80 *Listeria* isolates from Italian GSs showed a composition of 60% *L. innocua*, 30% *L. monocytogenes*, and 10% *L. seeligeri* [49]. Consistent with our results, all *Listeria*-positive GSs in other reports were of poor quality, with pH values ranging from 5.78 to 5.89 [59].

*Clostridia* reduce the nutritional value of silage by breaking down proteins and producing ammonia, thereby altering milk quality through the presence of biogenic amines. The presence of *Clostridia* in silage is primarily linked to soil contamination from manure and animal droppings. Consequently, GSs typically carry a higher load of ASFB than maize silages due to lower mowing heights, greater soil contamination, and slower acidification. This pattern was reflected in our findings, as the prevalence of ASFB in GSs was slightly higher than the 81% previously reported for MSs [30]. According to scientific recommendations, the maximum count of *Clostridia* spores in silage should not exceed 5 × 10^3^ cfu/g [63,64]. Based on this threshold, 73.7% of the GSs showed an acceptable level of ASFB, while 26.3% of the samples revealed dangerously high contamination. Furthermore, over 53% of the GSs exceeded 2 log_10_ cfu/g of ASFB, a value considered the threshold for concern. Other reports on GSs have shown average *Clostridia* counts of 3.22 log_10_ MPN/g, 3.3 log_10_ cfu/g, and between 2.1 and 6.1 log_10_ spores/g [65,66,67]. Further studies reported values of 5 × 10^3^ spores/g, ranging from below 10^2^ to 10^7^ cfu/g, and 3.6 to 5.24 log_10_ cfu/g in wilted grass silages [9,68,69]. Additionally, only one in five tested GSs had a pH value below 4.5, a threshold generally considered necessary to keep ASFB under control. Similar to the *Clostridia* genus, the prevalence of *C. perfringens* in GSs was higher than in MSs, where fewer than 25% of samples were *C. perfringens*-positive [30]. Significantly lower isolation rates were recorded in Germany, where only 2.7% (11/410) of grass silages were positive for *C. perfringens* [70]. Guidelines from Australia and New Zealand recommend a count below 100 *C. perfringens* spores per gram in GS, with an upper limit of 70,000 spores/g [71]. This recommendation was met by 93.7% of the silages tested, and no forage exceeded the upper limit. Furthermore, the estimated mean and median in our study were lower than the Australian results, which recorded 4000–6500 and 150–350 spores/g, respectively. In contrast, another Polish study reported a lower level of *C. perfringens* contamination in GSs than our findings, with values ranging from 0.25 to 1.48 log_10_ cfu/g [72].

*C. perfringens* type A was found to be overwhelmingly predominant among the isolates of this species, while toxotypes D and E were detected at much lower frequencies in the tested GSs. These results align with current knowledge, describing *C. perfringens* type A as an opportunistic pathogen. It commonly resides in the gut microbiota of healthy individuals, as well as in soil and rotting vegetation. Meanwhile, *C. perfringens* types B–G are dangerous, strict pathogens. A key finding of this study is the homogeneity of isolates from GSs with correct pH compared to their incorrect pH counterparts. This divergence highlights the critical role of successful lactic acid fermentation in inhibiting fastidious pathogens. A rapid drop in silage pH creates an inhospitable environment for strictly pathogenic strains of *C. perfringens*. Conversely, poor fermentation and insufficient acidification allow for the growth of a wider array of *C. perfringens* toxotypes. The presence of toxotypes D and E in poorly preserved silage poses a serious concern because *C. perfringens* type D causes enterotoxemia (pulpy kidney disease) in small ruminants and calves. Meanwhile, *C. perfringens* type E is an emerging pathogen associated with hemorrhagic enteritis in calves and lambs. In addition, our study estimated that over 45% of *C. perfringens* isolates were *cpb2* gene-positive in GSs, whereas a German study reported a lower prevalence, ranging around 23% [70]. Notably, the current study demonstrates a significantly higher prevalence of β2-toxigenic *C. perfringens* strains in silages with incorrect pH compared to those with correct pH. This suggests that poor ensiling conditions may selectively favor the growth of β2-toxigenic *C. perfringens* strains. This phenomenon is observed for the second time in our research, having been recorded first in MSs and now in GSs [30].

Although the occurrence of *C. botulinum* spores in silage is extremely rare, poultry manure should not be applied to any land designated for silage production. Despite this, poultry litter was applied to grass intended for ensiling on two out of the 160 surveyed farms. Bovine botulism is most frequently caused by toxin types C and D, or their mosaic variants, even though types A and B have also been reported [73]. The presence of *C. botulinum* type B in GSs necessitates vigilance, as botulism is a rare but often fatal intoxication classified as a foodborne zoonosis. Outbreaks of type B botulism in cattle linked to grass silage consumption have been previously documented [74]. In such cases, *C. botulinum* type B from the feces of affected cows can contaminate the farm environment, including manure, fertilized meadows, and fresh grass. This creates a cycle of reinfection in the herd and poses a risk of contaminating the resulting milk and meat products. This is especially hazardous because *C. botulinum* type B represents one of the most common causes of human botulism. Therefore, in line with the One Health approach to bovine-derived food production, the occurrence of this pathogen in animal feed directly affects the safety of the entire food supply chain.

While *Campylobacter* species can colonize the intestines of healthy cattle, and human campylobacteriosis outbreaks have been linked to raw milk and dairy products, we did not detect the pathogen in any of the GSs. Our results are consistent with findings from other authors who also reported an absence of *Campylobacter* spp. in GSs [59].

Even though most members of the *Enterobacteriaceae* family found in silages are non-pathogenic, their presence negatively affects the growth of LAB, silage palatability, and nutritional value. *Enterobacteriaceae* are sensitive to low pH; their counts usually decline sharply when the pH drops below 4.5, although growth has still been observed at pH 4.4. In this study, over 90% of the tested GSs did not exceed 1 log_10_ cfu/g. Other researchers have either not detected *Enterobacteriaceae* in GSs or reported levels of 3.5 log_10_ cfu/g on day 66 of ensiling, which became undetectable by day 99 in Ireland, as well as 5.27 log_10_ cfu/g in Italy and 6.2 log_10_ cfu/g in another Irish study [43,52,75,76,77]. Furthermore, fecal contamination of GSs, as estimated by coliform bacteria counts, has been reported to range from 4 to 5.80 log_10_ cfu/g [72].

While *Salmonella* spp. are relatively common on dairy farms, this pathogen is rarely found in silage [78]. A well-established fermentation process resulting in a final pH below 3.8 effectively eradicates the pathogen from the forage. Consequently, the presence of *Salmonella* spp. in silage indicates either poor fermentation or secondary contamination during aerobic exposure or feed-out. Previous studies have demonstrated an absence of *Salmonella* spp. or their presence at levels around 1.48 log_10_ cfu/g [72]. In our study, the presence of *Salmonella* spp. and *E. coli* in positive GS samples was favored by an exceptionally high pH and was preceded by the fertilization of grasses with manure. These circumstances pose a significant risk and align with the described ruminant salmonellosis outbreak due to silage, which was diagnosed when the silage crops were derived from a field where *Salmonella*-positive organic fertilizers had been spread [79].

Within the *Enterobacteriaceae* family, *E. coli* is the second most notorious species found in silages, primarily due to Shiga toxin-producing strains, well-known foodborne pathogens that cause severe hemorrhagic colitis and hemolytic uremic syndrome. The primary sources of *E. coli* contamination in GSs are manure or irrigation water. This bacterium can tolerate low-pH conditions and may exhibit increased acid resistance after adapting to a low-pH growth environment. However, a rapid drop in pH has been shown to eliminate *E. coli* in silage; consequently, the microorganism is typically not detected in GSs with a correct, low pH. Other studies have reported *E. coli* levels reaching up to 4.7 log_10_ cfu/g on day 66 of ensiling, dropping to undetectable levels by day 99, or reaching up to 6.2 log_10_ cfu/g [43,77]. Our findings are similar to other Polish and Irish studies, where *E. coli* counts ranged from not detected to an average of 1.75 log_10_ cfu/g [72,75].

The role of *B. cereus* as a critical spoilage organism in pasteurized dairy products stems from the ability of its spores to traverse the cattle digestive tract, thereby leading to the contamination of the farm environment [9]. Such transmission is highly correlated with the feed, as silages have been reported to contain substantial loads ranging up to 10^6^ *B. cereus* spores/g and 10^8^ other *Bacillus* spores/g [9,80]. The presence of *B. cereus* spores in silage originates mainly from soil and animal manure. Maize silage generally exhibits lower concentrations of total aerobic spores than GS (10–10^3^ cfu/g versus 10^2^–10^5^ cfu/g), as grass is more susceptible to soil or fecal contamination during harvesting [81]. In the surveyed GSs, we recorded lower levels of *B. cereus* contamination, ranging from 10^1^ to 10^3^ cfu/g. Considering that the recommended limit of *B. cereus* spores in feed is below 3 log_10_ spores/g, 91.25% of the analyzed silages were compliant [82]. Our results are consistent with other findings, where *B. cereus* spore concentrations reached 2.4 ± 0.07 log_10_ per gram in 117 mixed corn and grass silage samples [82]. A concerning observation is that the prevalence of *B. cereus* in the tested GSs was twice as high as that in the previously reported MSs [30]. All species of the *Bacillus* genus identified in forages are facultative anaerobes and have been linked to the later stages of aerobic silage deterioration. Food poisoning and gastrointestinal diseases are well-known to be caused by *B. cereus*, the primary pathogenic species. Although *B. thuringiensis* is not deemed pathogenic to humans, some strains can infect immunocompromised individuals. Meanwhile, *B. weihenstephanensis* and *B. mycoides* exhibit low pathogenicity, though they are well-documented agents of pasteurized milk spoilage. The lower count of *B. cereus* in inoculated GSs observed in our study is consistent with previous findings. In this study, the *Bacillus* spore count was higher in non-inoculated silage (average of 3.18 log cfu/g) than in silage inoculated with SiloSolve^®^ FC (average of 2.74 log cfu/g; 50:50 ratio of *L. buchneri* DSM22501 and *L. lactis* O224 DSM11037) [76]. Moreover, a negative correlation between LAB count and *B. cereus* burden confirmed hardly any growth of *B. cereus* in GSs where LAB vigorously developed. In turn, a negative correlation was observed between temperature and both the count and prevalence of *B. cereus* in GSs, which contrasts with our earlier findings reported for MSs, as well as other observations indicating that high storage temperatures stimulate the growth of *Bacillus* species in grass silage [9,30]. A positive correlation was noted between organic fertilization and the *B. cereus* load of GSs. Specifically, fertilizing with manure increases the count of *Bacillus* spores on the crop, which in turn increases the number of spores in the silage, raising its pH value.

Prior to ensiling, the forage microbiome exhibits significantly greater abundance and diversity compared to post-ensiling stages, as anaerobic fermentation and low pH inhibit most microorganisms. Nevertheless, the high moisture content of GS provides a suitable environment for microbial proliferation. Hygienic indicators, such as TPC or AMB, are commonly employed to assess sanitary quality, sensory acceptability, and compliance with good manufacturing practices. These indicators reflect the initial quality and handling history of the forage, as well as the conditions during ensiling and storage. Furthermore, they serve to estimate shelf-life, predict impending sensory degradation, and evaluate the likelihood of pathogen and toxin occurrence, despite the absence of a direct correlation with the overall biohazard burden. When interpreting AMB and TPC in fermented feeds such as silage, a high microbial load is inherently expected. In the present study, TPC ranged from 2 to 9.7 log_10_ cfu/g across all tested silages and from 2 to 8.2 log_10_ cfu/g in silages with a correct pH. In turn, the AMB count ranged from 1.6 to 9.5 log_10_ cfu/g across all tested grass silages and from 2.6 to 7.2 log_10_ cfu/g in samples with a correct pH. In comparison, other researchers have reported levels of 7.0 ± 1.0 log_10_ cfu/g or 7.1–8.3 log_10_ cfu/g after 66 days of ensiling, and this count remained relatively stable at approximately 6.0 ± 1.0 log_10_ cfu/g throughout the fermentation process [43,77]. When the German GSs were analyzed, the majority of samples in each region were classified as Quality Standard 1 (QS = 1) based on reference ranges established by the Association of German Agricultural Inspection and Research Institutes [48]. In the East and South regions, this accounted for around 80% of GSs, and in the North region, almost 70%. However, in each region, 10% of the GSs tested were found to be of insufficient microbiological quality (QS = 4), and in the North region, this figure was almost 20%. Additionally, the microbiological analysis of GSs from all over Germany showed that 70–80% of forages were of good, fault-free quality (QS = 1) compared to 50–70% of MSs.

Filamentous fungi are distributed worldwide and form symbiotic or pathogenic relationships with many grasses. The occurrence of fungi in silage is usually restricted to the surface layers and often indicates poor sealing and compaction. However, in baled GSs, air ingress can lead to fungal development throughout the inside of the bale. This is hazardous, as many molds are capable of producing mycotoxins, which decrease the nutritional value and palatability of the forage while reducing feed intake. In severe cases, these toxins can lead to respiratory problems, allergies, abnormal ruminal fermentation, increased abortions, hormonal imbalances, and suppressed immune function [9]. Moreover, inhalation or intake of fungal propagules may cause diseases collectively known as mycosis. Although the total fungal count in silage should not be used as a direct indicator of mycotoxin presence, a count exceeding 6 log_10_ cfu/g is associated with aerobically spoiled, non-feedable silage [63,83]. In contrast, according to good manufacturing practices for animal feed, a fungal count of 4 log_10_ cfu/g represents the maximum recommended threshold [84]. Consequently, a count below this level reflects good-quality silage. The fungal count in surveyed Polish GSs ranged from 1 to 7.8 log_10_ cfu/g. In light of the above reference values, our study revealed that 10.6% of the GSs exceeded 6 log_10_ cfu/g of fungi, while 73.1% fell below 4 log_10_ cfu/g. The remaining 16.3% of the tested silages ranged between 4 and 6 log_10_ cfu/g. Other reports have demonstrated fungal counts in GSs ranging from 3.8 to 6.8 log_10_ cfu/g and <2.0 to 4.9 log_10_ cfu/g in the Netherlands [13,42], 2.31 to 3.09 log_10_ cfu g^−1^ and 2.54 to 3.74 log_10_ cfu g^−1^ in Serbia [85,86], and 4.60 to 6.30 log_10_ cfu/g in another Polish study [72]. The fungal count in silage is significantly reduced by the addition of an inoculant, which substantially lowers the silage pH, as demonstrated in our study by a correlation coefficient of −0.19. Based on the above findings, it is worth considering fungal enumeration alongside pH value in routine silage evaluation, since it is known that pH is a key factor driving fungal growth in silage, as reflected by the positive correlation between these variables in our study. Lowering the silage pH through inoculation also reduces the LAB count. This is confirmed by literature data, where the LAB level in experimentally inoculated GS was 8.0 log_10_ cfu/g, compared to 9.59 log_10_ cfu/g in the uninoculated control silage [76]. We recorded a similar relationship in our study, indicated by a negative correlation of −0.12 between silage inoculation and the LAB count. Moreover, a negative correlation was found between GS inoculation and the counts of *Clostridia*, *B. cereus*, *Enterobacteriaceae*, *E. coli*, fungi, AMB, and TPC, as well as the occurrence of botulism. This is consistent with observations where the use of LAB additives improved fermentation quality and modified the bacterial profiles of GSs [37]. Similarly, adding biological ensiling additives to experimental silages has shown reduced counts of *Enterobacteriaceae*, spore-forming, and anaerobic microorganisms, as well as yeasts, while improving fermentation quality compared to the control group [87]. The minimum LAB count required to suppress *Clostridia* was found to be 100,000 cfu/g, which corresponds to 5 log_10_ cfu/g [88]. This requirement was met by 58.8% of the tested GS samples. Generally, the total LAB population in silage hovers around 7 log_10_ cfu/g, though previous studies indicate that a higher inoculation rate can lead to a lower overall count [9]. In our study, LAB counts in nationwide GS samples varied widely, spanning from 1.0 to 8.9 log_10_ cfu/g. Specifically, 55.6% of the GS samples fell below 6 log_10_ cfu/g, while only 5.0% surpassed 8 log_10_ cfu/g. Notably, 5.6% of the silages had a LAB count under 2 log_10_ cfu/g, exhibiting a pH range of 3.8–6.8. The low LAB levels detected in certain forages may reflect a bacterial response to adverse environmental conditions, which can induce a dormant state. In comparison, the literature data show diverse LAB populations across different studies. Reported counts range from 1.88 × 10^2^–7.55 × 10^3^ cfu/g and 3.7–5.09 log_10_ cfu/g in Poland [54,89], 3.93–5.55 log_10_ cfu/g in Brazil [90], 5.2–7.1 log_10_ cfu/g and 6.5–7.6 in Ireland [43,77] and 8.0–9.59 log_10_ cfu/g in Italy [76]. These global variations, coupled with our findings, underscore that silage microbiota dynamics are highly dependent on regional harvesting conditions, forage management, and physiological bacterial adaptations.

Even though freezing may slightly affect microbial viability, logistical constraints inherent to a national-scale study rendered this the only viable approach. Due to the significant geographical distances between sampling sites and the laboratory, standard refrigeration was insufficient to guarantee sample stability. To ensure data integrity, all silage samples underwent an identical freezing protocol; thus, any potential attenuation or shift in microbial loads was uniform across the dataset. Furthermore, this study is limited by the lack of traceability concerning the transfer of silage-borne biological and chemical hazards through dairy cattle into raw milk. This includes the monitoring of mycotoxins, despite the high fungal counts detected in certain samples. Additionally, the characterization of microbiological quality and pH dynamics is limited by the absence of data regarding harvest moisture content and organic acid profiles. Future investigations must integrate these variables to provide a more comprehensive analysis of silage hygiene and preservation quality.

It is worth noting that while the samples analyzed in this study were collected over a decade ago, the fundamental production practices, harvesting technologies, and standard silage additives used for grass ensiling in Poland have remained highly consistent over the last 10 years. Therefore, these findings remain fully relevant and representative of contemporary agricultural standards in Central Europe.

## 5. Conclusions

Feed represents one of the largest economic inputs in cattle production, serving as a critical determinant of both herd health and farm profitability. Although silages represent a cornerstone of modern dairy and beef cattle diets, their quality is frequently evaluated without accounting for pathogenic microorganisms. However, substandard or contaminated silages can harbor pathogens that impair animal performance, induce bovine diseases, and pose substantial threats to human health. This study confirms that GS is highly vulnerable to poor fermentation and microbial contamination. The consistency of our findings across samples of various maturities underscores that the crucial factor for silage safety is the initial stabilization of the material. In properly fermented GS, the pH drops to pathogen-inhibiting levels within two weeks, and this protective acidity remains stable in well-wrapped bales for up to three years. Consequently, bales stored for one month and those kept for extended periods under anaerobic conditions offer a substantially similar environment for pathogen inhibition. Therefore, implementing good manufacturing practices and a wider adoption of biological inoculants are crucial steps required to secure feed safety.

As silage constitutes a critical vector for pathogen introduction into the food supply chain, eliminating contamination is paramount. The sample size and microbial characterization of the GSs investigated herein enhance the understanding of forage hygiene, providing empirical data to inform scientific risk assessment and management strategies regarding microbial prevalence and quantification. Maintaining strictly anaerobic conditions within a pH spectrum of 3.4–4.3 suppresses the proliferation of facultatively anaerobic pathogens, including *E. coli*, *L. Monocytogenes*, and *B. cereus*. However, anaerobic environments at pH 4.1 remain susceptible to *C. butyricum* activity (the primary driver of butyric fermentation), while pH levels above 4.5 promote the growth of botulinum toxin-producing *Clostridia*. Recommended microbiological limits for GS comprise ≤2 cfu/g for both *Clostridia* and *Bacillus*, <50 cfu/g for *C. Perfringens*, and <4 cfu/g for total fungal counts. Additionally, the administration of inoculants improves hygienic quality and safety margins, particularly under high risks of microbial spoilage, deficient epiphytic microbiota on the standing crop, or elevated moisture content.

## Figures and Tables

**Table 1:** Distribution of microbial counts (log_10_ cfu/g) in GSs of correct pH and descriptive statistics.

Microorganism	1 log_10_	2 log_10_	3 log_10_	4 log_10_	5 log_10_	6 log_10_	7 log_10_	8 log_10_	9 log_10_	Range (log_10_ cfu/g)	Mean Value (log_10_ cfu/g)	Median Value (log_10_ cfu/g)
	% of Samples											
TPC	0	3.6	25.0	17.8	25.0	21.4	3.6	3.6	0	2–8.2	5	5
AMB	0	7.1	21.4	35.7	17.8	14.3	3.6	0	0	2.6–7.2	4.7	4.6
Fungi	67.8	10.7	10.7	3.6	3.6	3.6	0	0	0	1–6.2	2	1.1
LAB	10.7	7.1	17.8	25.0	7.1	25.0	7.1	0	0	1–7.3	4.5	4.5
*Enterobacteriaceae*	100	0	0	0	0	0	0	0	0	1.0	1.0	1.0
*E. coli*	100	0	0	0	0	0	0	0	0	1.0	1.0	1.0
*Clostridium*	21.4	25.0	21.4	25.0	7.1	0	0	0	0	1–5	2.7	3.0
*C. perfringens*	92.8	7.1	0	0	0	0	0	0	0	1–2.4	1.1	1.0
*B. cereus*	75	21.4	3.6	0	0	0	0	0	0	1–3.5	1.5	1.0

**Table 2:** Distribution of microbial counts (log_10_ cfu/g) in GSs of incorrect pH and descriptive statistics.

Microorganism	1 log_10_	2 log_10_	3 log_10_	4 log_10_	5 log_10_	6 log_10_	7 log_10_	8 log_10_	9 log_10_	Range (log_10_ cfu/g)	Mean Value (log_10_ cfu/g)	Median Value (log_10_ cfu/g)
	% of Samples											
TPC	0	5.3	12.9	16.7	22.0	19.7	15.9	6	1.5	2.2–9.7	5.7	5.7
AMB	0.7	4.5	17.4	16.7	23.5	16.7	15.1	3.8	1.5	1.6–9.5	5.5	5.5
Fungi	42.4	15.9	11.4	10.6	7.6	3.0	9.0	0	0	1–7.8	3.0	2.2
LAB	4.5	5.3	13.6	13.6	15.9	28.0	12.1	6.8	0	1–8.9	5.5	5.7
*Enterobacteriaceae*	88.6	2.3	1.5	4.5	1.5	1.5	0	0	0	1–6.3	1.4	1.0
*E. coli*	98.5	1.5	0	0	0	0	0	0	0	1–2.4	1.0	1.0
*Clostridium*	16.7	30.3	28.0	12.9	12.1	0	0	0	0	1–5	2.7	3.0
*C. perfringens*	93.3	4.5	1.5	0	0	0	0	0	0	1–3.6	1.1	1.0
*B. cereus*	66.7	23.5	9.8	0	0	0	0	0	0	1–3.7	1.6	1.0

**Table 3:** Distribution of microbial counts (log_10_ cfu/g) in total GSs and descriptive statistics.

Microorganism	1 log_10_	2 log_10_	3 log_10_	4 log_10_	5 log_10_	6 log_10_	7 log_10_	8 log_10_	9 log_10_	Range (log_10_ cfu/g)	Mean Value (log_10_ cfu/g)	Median Value (log_10_ cfu/g)
	% of Samples											
TPC	0	4.7	14.2	16.0	21.3	18.9	13.0	5.3	1.2	2–9.7	5.6	5.5
AMB	0.6	5.0	18.1	20.0	22.5	16.3	13.1	3.1	1.3	1.6–9.5	5.3	5.4
Fungi	46.9	15.0	11.3	9.4	6.9	3.1	7.5	0	0	1–7.8	2.8	2.1
LAB	5.6	5.6	14.4	15.6	14.4	27.5	11.3	5.6	0	1–8.9	5.3	5.7
*Enterobacteriaceae*	90.6	1.9	1.3	3.8	1.3	1.3	0	0	0	1–6.3	1.3	1.0
*E. coli*	98.8	1.3	0	0	0	0	0	0	0	1–2.4	1.0	1.0
*Clostridium*	17.5	29.4	26.9	15.0	11.3	0	0	0	0	1–5	2.7	3.0
*C. perfringens*	93.8	5.0	1.3	0	0	0	0	0	0	1–3.6	1.1	1.0
*B. cereus*	68.1	23.1	8.8	0	0	0	0	0	0	1–3.7	1.6	1.0

**Table 4:** The differences in microbial load of inoculated and non-inoculated and organically fertilized and non-fertilized GSs.

Microorganism	Inoculated GSs			Non-Inoculated GSs			Fertilized GSs			Non-Fertilized GSs		
	Range (log_10_ cfu/g)	Mean Value (log_10_ cfu/g)	Median Value (log_10_ cfu/g)	Range (log_10_ cfu/g)	Mean Value (log_10_ cfu/g)	Median Value (log_10_ cfu/g)	Range (log_10_ cfu/g)	Mean Value (log_10_ cfu/g)	Median Value (log_10_ cfu/g)	Range (log_10_ cfu/g)	Mean Value (log_10_ cfu/g)	Median Value (log_10_ cfu/g)
TPC	2–9.4	5.3	5.2	2.2–9.7	5.7	5.6	2.4–8.5	5.5	5.4	2–9.7	5.7	5.8
AMB	2.2–8.4	5.0	5.1	1.6–9.5	5.4	5.5	2.3–9.5	5.4	5.5	1.6–9.5	5.3	5.4
Fungi	1–7.5	2.3	1.2	1–7.8	3.0	2.3	1–7.5	2.5	2.0	1–7.8	3.0	2.1
LAB	1–8.5	4.9	5.1	1–8.9	5.4	5.7	1–8.5	5.2	5.5	1–8.9	5.4	5.7
*Enterobacteriaceae*	1–6.3	1.3	1	1–6.2	1.3	1	1–6.3	1.2	1	1–6.2	1.4	1
*E. coli*	1–1.8	1.0	1	1–2.4	1.0	1	1–1.5	1.0	1	1–2.4	1.1	1
*Clostridium*	1–5	2.4	2	1–5	2.8	3	1–5	2.9	3	1–5	2.6	3
*C. perfringens*	1–2.2	1.1	1	1–3.6	1.1	1	1–3.6	1.1	1	1–2.5	1.1	1
*B. cereus*	1–3.6	1.6	1	1–3.7	1.6	1	1–3.7	1.7	1	1–3.6	1.6	1

## Data Availability

The original contributions presented in this study are included in the article. Further inquiries can be directed to the corresponding author.

## References

[1] Wilkinson J.M., Rinne M. (2018). Highlights of progress in silage conservation and future perspectives. Grass Forage Sci..

[2] Roth A. (2025). Uniting dairy excellence & ambition. Dairy Focus.

[3] Butler G., Nielsen J.H., Slots T., Seal C., Eyre M.D., Sanderson R., Leifert C. (2008). Fatty acid and fat-soluble antioxidant concentrations in milk from high- and low-input conventional and organic systems: Seasonal variation. J. Sci. Food Agric..

[4] Wilkinson J.M., Davies D.R. (2012). The aerobic stability of silage: Key findings and recent developments. Grass Forage Sci..

[5] Lád F., Kubát V., Kadlec J., Čermák B., Kobes M. (2011). The effects of biological additives on the fermentation process and fibre composition of farm-scale grass silages. J. Agrobiol..

[6] Liu J., Seiji K., Junjiro S., Masahiko O., Yasushi A. (1986). The nutritive values of grass, corn and rice silages fed to sheep at different levels. J. Fac. Agric..

[7] Radkowski A., Kuboń M. (2013). Quality and nutritional value of silage made of meadow grass mowed with and without conditioners. Agric. Eng..

[8] Dolci P., Tabacco E., Cocolin L., Borreani G. (2011). Microbial dynamics during aerobic exposure of corn silage stored under oxygen barrier or polyethylene films. Appl. Environ. Microbiol..

[9] Pahlow G., Muck R.E., Driehuis F., Oude Elferink S.J.W.H., Spoelstra S.F., Buxton D.R., Muck R.E., Harrison J.H. (2003). Microbiology of ensiling. Silage Science and Technology.

[10] Driehuis F., Wilkinson J.M., Jiang Y., Ogunade I., Adesogan A.T. (2018). Silage review: Animal and human health risks from silage. J. Dairy Sci..

[11] Queiroz O.C.M., Ogunade I.M., Weinberg Z., Adesogan A.T. (2017). Silage review: Foodborne pathogens in silage and their mitigation by silage additives. J. Dairy Sci..

[12] Muck R.E. (2010). Silage microbiology and its control through additives. Rev. Bras. Zootec..

[13] Vissers M.M.M., Driehuis F., Te Giffel M.C., De Jong P., Lankveld J.M.G. (2007). Concentrations of Butyric Acid Bacteria Spores in Silage and Relationships with Aerobic Deterioration. J. Dairy Sci..

[14] Gessler F., Böhnel H. (2006). Persistence and mobility of a *Clostridium botulinum* spore population introduced to soil with spiked compost. FEMS Microbiol. Ecol..

[15] Colditz I.G. (2002). Effects of the immune system on metabolism: Implications for production and disease resistance in livestock. Livest. Prod. Sci..

[16] Rubin B.E.R., Gibbons S.M., Kennedy S., Hampton-Marcell J., Owens S., Gilbert J.A. (2013). Investigating the impact of storage conditions on microbial community composition in soil samples. PLoS ONE.

[17] Woodard K.R., Prine G.M., Bates D.B. (1991). Silage characteristics of elephantgrass as affected by harvest frequency and genotype. Agron. J..

[18] (2002). Microbiology of Food and Animal Feeding Stuffs—Horizontal Method for the Detection of Salmonella spp.

[19] (1994). Animal Feedingstuffs. Requirements and Microbiological Examination.

[20] (1999). Microbiology of Food and Animal Feeding Stuffs—Horizontal Method for the Detection and Enumeration of Listeria monocytogenes. Part 1. Detection Method.

[21] (2006). Microbiology of Food and Animal Feeding Stuffs—Horizontal Method for Detection and Enumeration of Campylobacter spp. Part 1: Detection Method.

[22] (2013). Microbiology of the Food Chain—Horizontal Method for the Enumeration of Microorganisms. Part 1. Colony Count at 30 Degrees C by the Pour Plate Technique.

[23] (1997). Meat and Meat Preparations—Microbiological Tests—Detection of Anaerobic Spore-Forming Bacteria and Anaerobic Spore-Forming Sulfate(IV)-Reducing Bacteria.

[24] (2004). Microbiology of Food and Animal Feeding Stuffs—Horizontal Method for the Detection and Enumeration of Enterobacteriaceae. Part 2. Colony-Count Technique.

[25] (2001). Microbiology of Food and Animal Feeding Stuffs—Horizontal Method for the Enumeration of Beta-Glucuronidase-Positive Escherichia coli. Part 2. Colony-Count Technique at 44 °C Using 5-Bromo-4-chloro-3-indolyl beta-D-glucuronide.

[26] (2004). Microbiology of Food and Animal Feeding Stuffs—Horizontal Method for the Enumeration of Presumptive Bacillus cereus. Colony-Count Technique at 30 Degrees C.

[27] (2004). Microbiology of Food and Animal Feeding Stuffs—Horizontal Method for the Enumeration of Clostridium perfringens. Colony-Count Technique.

[28] (1998). Microbiology of Food and Animal Feeding Stuffs—Horizontal Method for the Enumeration of Mesophilic Lactic Acid Bacteria. Colony-Count Technique at 30 Degrees C.

[29] (1999). Microbiology of Food and Animal Feeding Stuffs—Horizontal Method for the Enumeration of Coagulase-Positive Staphylococci (Staphylococcus aureus and Other Species). Part 2: Technique Using Rabbit Plasma Fibrinogen Agar Medium.

[30] Kukier E., Bocian Ł., Pytka M. (2026). Microbiological Quality of Maize Silage in Relation to Agricultural Practices: A Four-Year Study. Foods.

[31] (2017). Microbiology of the Food Chain—Preparation of Test Samples, Initial Suspension and Decimal Dilutions for Microbiological Examination—Part 4: Specific Rules for the Preparation of Miscellaneous Products.

[32] Baums C.G., Schotte U., Amtsberg G., Goethe R. (2004). Diagnostic multiplex PCR for toxin genotyping of *Clostridium perfringens* isolates. Vet. Microbiol..

[33] Kukier E., Kwiatek K. (2010). Occurrence of *Clostridium perfringens* in food chain. Bull. Vet. Inst. Pulawy.

[34] Raphael B.H., Andreadis J.D. (2007). Real-time PCR detection of the nontoxic nonhemagglutinin gene as a rapid screening method for bacterial isolates harboring the botulinum neurotoxin (A–G) gene complex. J. Microbiol. Methods.

[35] De Medici D., Anniballi F., Wyatt G.M., Lindstrom M., Messelhausser U., Aldus C.F., Delibato E., Korkeala H., Peck M.W., Fenicia L. (2009). Multiplex PCR for detection of botulinum neurotoxin-producing Clostridia in clinical, food, and environmental samples. Appl. Environ. Microb..

[36] Eikmeyer F., Köfinger P., Poschenel A., Jünemann S., Zakrzewski M., Heinl S., Mayrhuber E., Grabherr R., Pühler A., Schwab H. (2013). Metagenome analyses reveal the influence of the inoculant Lactobacillus buchneri CD034 on the microbial community involved in grass ensiling. J. Biotechnol..

[37] Franco M., Tapio I., Pirttiniemi J., Stefański T., Jalava T., Huuskonen A., Rinne M. (2022). Fermentation quality and bacterial ecology of grass silage modulated by additive treatments, extent of compaction and soil contamination. Fermentation.

[38] Kleinschmit D.H., Kung L. (2006). A meta-analysis of the effects of Lactobacillus buchneri on the fermentation and aerobic stability of corn and grass and small-grain silages. J. Dairy Sci..

[39] Relun A., Dorso L., Douart A., Chartier C., Guatteo R., Mazuet C., Assié S. (2017). A large outbreak of bovine botulism possibly linked to a massive contamination of grass silage by type D/C *Clostridium botulinum* spores on a farm with dairy and poultry operations. Epidemiol. Infect..

[40] Terentjeva M., Šteingolde Ž., Meistere I., Elferts D., Avsejenko J., Streikiša M., Gradovska S., Alksne L., Ķibilds J., Bērziņš A. (2021). Prevalence, genetic diversity and factors associated with distribution of *Listeria monocytogenes* and other *Listeria* spp. in cattle farms in Latvia. Pathogens.

[41] Septian F., Kardaya D., Astuti W.D. (2011). Evaluasi kualitas silase limbah sayuran pasar yang diperkaya dengan berbagai aditif dan bakteri asam laktat. J. Pertan..

[42] Driehuis F. (2013). Silage and the safety and quality of dairy foods: A review. Agric. Food Sci..

[43] Esteves E., Whyte P., Gupta T.B., Bolton D. (2021). The survival of blown pack spoilage associated *Clostridium estertheticum* and *Clostridium gasigenes* spores during the ensiling of grass. FEMS Microbes.

[44] Franco M., Rinne M. (2023). Dry matter content and additives with different modes of action modify the preservation characteristics of grass silage. Fermentation.

[45] Jonsson A. (1991). Growth of *Clostridium tyrobutyricum* during fermentation and aerobic deterioration of grass silage. J. Sci. Food Agric..

[46] Keady T. (2012). High feed value grass silage: Its importance and production. Ir. Grassl. Assoc. J..

[47] Kung L., Shaver R.D. (2001). Interpretation and use of silage fermentation analysis reports. Focus Forage.

[48] Litjens I. (2020). Untersuchungen zur Silagequalität—Siliererfolg/Hygienestatus und Futterwert—Auf Milchkuhbetrieben in Deutschland. https://elib.tiho-hannover.de/receive/tiho_mods_00003632.

[49] Nucera D.M., Grassi M.A., Morra P., Piano S., Tabacco E., Borreani G. (2016). Detection, identification, and typing of *Listeria* species from baled silages fed to dairy cows. J. Dairy Sci..

[50] O’Brien M., O’Kiely P., Forristal P.D., Fuller H.T. (2006). A note on sampling baled grass silage for fungal propagules. J. Anim. Feed Sci..

[51] O’Kiely P., Moloney A.P. (1994). Silage characteristics and performance of cattle offered grass silage made without an additive, with formic acid or with a partially neutralised blend of aliphatic organic acids. Ir. J. Agric. Food Res..

[52] Pahlow G. (1984). O 2-abhängige Veränderungen der Mikroflora in Silagen mit Lactobacterienzusatz. Landwirt. Forsch. Kongressband.

[53] Perry C.M., Donnelly C.W. (1990). Incidence of *Listeria monocytogenes* in silage and its subsequent control by specific and nonspecific antagonism. J. Food Prot..

[54] Wróbel B. (2012). Jakość kiszonek z runi łąkowej z dodatkiem biologicznych stymulatorów fermentacji. Woda-Środowisko-Obsz. Wiej..

[55] Vaičiulienė G., Bakutis B., Jovaišienė J., Falkauskas R., Gerulis G., Kerziene S., Baliukoniene V. (2021). Prevalence of mycotoxins and endotoxins in total mixed rations and different types of ensiled forages for dairy cows in Lithuania. Toxins.

[56] Vilar M.J., Yus E., Sanjuan M.L., Diequez F.J., Rodriquez-Otero J.L. (2007). Prevalence of and risk factors for *Listeria* species on dairy farms. J. Dairy Sci..

[57] Fulgueira C.L., Amigot S.L., Gaggiotti M., Romero L.A., Basílico J.C. (2007). Forage quality: Techniques for testing. Fresh Prod..

[58] Ryser E.T., Arimi S.M., Donnelly C.W. (1997). Effects of pH on distribution of *Listeria* ribotypes in corn, hay, and grass silage. Appl. Environ. Microbiol..

[59] Grant M.A., Eklund C.A., Shields S.C. (1995). Monitoring dairy silage for five bacterial groups with potential for human pathogenesis. J. Food Prot..

[60] Castro H., Jaakkonen A., Hakkinen M., Korkeala H., Lindström M. (2018). Occurrence, persistence, and contamination routes of *Listeria monocytogenes* genotypes on three Finnish dairy cattle farms: A longitudinal study. Appl. Environ. Microbiol..

[61] Çokal Y. (2014). The presence of *Listeria* species in dairy cattle farms in Bandırma province. Etlik Vet. Mikrobiyol. Derg..

[62] Husu J.R., Sivelä S.K., Rauramaa A.L. (1990). Prevalence of *Listeria* species as related to chemical quality of farm-ensiled grass. Grass Forage Sci..

[63] Doležal P., Dvořáček J., Loučka R. (2012). Konzervace krmiv a jejich využití ve výživě zvířat. Vydání: 1 Olomouc: Vydavatelství Baštan.

[64] Zeman L., Doležal P., Kopřiva A. (2006). Výživa a Krmení Hospodářských Zvířat.

[65] Zucali M., Bava L., Colombini S., Brasca M., Decimo M., Morandi S., Tamburini A., Crovetto G.M. (2015). Management practices and forage quality affecting the contamination of milk with anaerobic spore-forming bacteria. J. Sci. Food Agric..

[66] Custódio L., Morais G., Daniel J.L., Pauly T., Nussio L.G. (2016). Effects of chemical and microbial additives on *Clostridium* development in sugarcane (*Saccharum officinarum* L.) ensiled with lime. Grassl. Sci..

[67] Nadeau E., Rustas B.-O., Arnesson A., Swensson C. Maize silage quality on Swedish dairy and beef farms. Proceedings of the 14th International Symposium Forage Conservation.

[68] Rammer C.H. (1996). Quality of grass silage infected with spores of *Clostridium tyrobutyricum*. Grass Forage Sci..

[69] Corporaal J., Steg A. (1990). Beinonstering, Kwaliteit En Voederwaardering Van Graskuil (Sampling, Fermentation Quality and Feed Evaluation of Grass Silage).

[70] Fohler S., Klein G., Hoedemaker M., Scheu T., Seyboldt C., Campe A., Jensen K.C., Abdulmawjood A. (2016). Diversity of *Clostridium perfringens* toxin-genotypes from dairy farms. BMC Microbiol..

[71] Goeser J. (2026). Mold, Yeast and *Clostridium perfringens* Guidelines for Agricultural Feeds and Total Mixed Rations.

[72] Zielińska K.J., Fabiszewska A.U., Wróbel B.J. (2016). An assessment of the microbiological and chemical quality of grass silages from selected organic farms. Res. Appl. Agric. Eng..

[73] Anniballi F., Fiore A., Löfström C., Skarin H., Auricchio B., Woudstra C., Bano L., Segerman B., Koene M., Båverud V. (2013). Management of animal botulism outbreaks: From clinical suspicion to practical countermeasures to prevent or minimize outbreaks. Biosecur. Bioterror..

[74] Kelch W.J., Kerr L.A., Pringle J.K., Rohrbach B.W., Whitlock R.H. (2000). Fatal *Clostridium botulinum* toxicosis in eleven Holstein cattle fed round bale barley haylage. J. Vet. Diagn. Investig..

[75] O’Kiely P., Byrne C., Bolton D. (2001). Survival of *Escherichia coli* 0157:H7 added to grass at ensiling and its influence on silage fermentation. IGC Proceedings, (1977–2023).

[76] Copani G., Milora N., Segura A., Witt K., Ghilardelli F., Sigolo S., Gallo A. Control of pathogenic *Escherichia coli* O157:H7 in contaminated grass silage with dual-purpose silage inoculant. Proceedings of the VI International Symposium on Forage Quality and Conservation (ISFQC).

[77] Nyambe S., Burgess C., Whyte P., O’Kiely P., Bolton D. (2017). The fate of verocytotoxigenic *Escherichia coli* C600φ3538(Δvtx2::cat) and its vtx2 prophage during grass silage preparation. J. Appl. Microbiol..

[78] Davison H.C., Sayers A.R., Smith R.P., Pascoe S.J., Davies R.H., Weaver J.P., Evans S.J. (2006). Risk factors associated with the *Salmonella* status of dairy farms in England and Wales. Vet. Rec..

[79] Ruoho O. (2007). An Epidemic of Salmonellosis Caused by Silage Containing Salmonella at a Dairy Farm.

[80] Te Giffel M.C. (1997). Isolation, Identification and Characterization of *Bacillus cereus* from the Dairy Environment. Ph.D. Dissertation.

[81] Te Giffel M.C., Wagendorp A., Herrewegh A., Driehuis F. (2002). Bacterial spores in silage and raw milk. Antonie Van Leeuwenhoek.

[82] Vissers M., Te Giffel M., Driehuis F., De Jong P., Lankveld J.M.G. (2007). Minimizing the level of *Bacillus cereus* spores in farm tank milk. J. Dairy Sci..

[83] Gotlieb A. Mycotoxins in Silage: A Silent Loss in Profits. https://www.northeastipm.org/ipm-in-action/ipmresources/resource-detail/?id=3788.

[84] (2009). Good Manufacturing Practices. Certification Scheme Animal Feed Sector, 2006. Version Marzo 2008.

[85] Bijelić Z., Krnjaja V., Tomić Z., Stojanović L., Ružić-Muslić D. (2009). Identification and quantification of fungi in grass-leguminous silage. Biotechnol. Anim. Husb..

[86] Bijelić Z., Krnjaja V., Stanković S., Muslić-Ružić D., Mandić V., Škrbić Z., Lukić M. (2017). Occurrence of moulds and mycotoxins in grass-legume silages influenced by nitrogen fertilization and phenological phase at harvest. Rom. Biotechnol. Lett..

[87] Mlejnková V., Fröhdeová M., Kalhotka L., Doležal P. (2014). Microbiological quality of experimental silages in combination with the addition of topsoil soil layer and ensiling additives. Acta Univ. Agric. Silvic. Mendel. Brun..

[88] Kaiser E., Weiss K. (1997). Fermentation process during the ensiling of green forage low in nitrate. 2. Fermentation process after supplementation of nitrate, nitrite, lactic-acid bacteria and formic acid. Arch. Anim. Nutr..

[89] Bodarski R., Stempniewicz R., Krzywiecki S., Krzyśko-Łupicka T., Słupczyńska M. Quality, microbiological status and aerobic stability of wilted grass-alfalfa silages made with different (chemical or microbiological) additives. Proceedings of the 11th International Scientific Symposium “Forage Conservation”.

[90] Mauro E., da Silva T.C., Oliveira Macedo C.H., Sena F. (2013). Lactic Acid Bacteria in Tropical Grass Silages. Lactic Acid Bacteria—R&D for Food, Health and Livestock Purposes.

